# Early epilepsy in children with Zika‐related microcephaly in a cohort in Recife, Brazil: Characteristics, electroencephalographic findings, and treatment response

**DOI:** 10.1111/epi.16444

**Published:** 2020-02-17

**Authors:** Maria Durce C. G. Carvalho, Ricardo A. A. Ximenes, Ulisses R. Montarroyos, Paula F. S. da Silva, Luciana P. A. Andrade‐Valença, Sophie H. Eickmann, Regina C. Ramos, Maria Ângela W. Rocha, Thalia V. B. de Araujo, Maria de Fátima P. M. de Albuquerque, Celina M. T. Martelli, Wayner V. de Souza, Elizabeth B. Brickley, Demócrito de B. Miranda‐Filho, on behalf of the Microcephaly Epidemic Research Group

**Affiliations:** ^1^ University of Pernambuco Recife Brazil; ^2^ Federal University of Pernambuco Recife Brazil; ^3^ Aggeu Magalhães Research Center Fiocruz Pernambuco Recife Brazil; ^4^ London School of Hygiene and Tropical Medicine London UK

**Keywords:** congenital Zika syndrome, epilepsy, microcephaly, seizure, Zika virus

## Abstract

**Objective:**

To estimate the incidence of epilepsy in children with Zika‐related microcephaly in the first 24 months of life; to characterize the associated clinical and electrographic findings; and to summarize the treatment responses.

**Methods:**

We followed a cohort of children, born during the 2015‐2016 Zika virus (ZIKV) epidemic in Brazil, with congenital microcephaly and evidence of congenital ZIKV infection on neuroimaging and/or laboratory testing. Neurological assessments were performed at ≤3, 6, 12, 15, 18, 21, and 24 months of life. Serial electroencephalograms were performed over the first 24 months.

**Results:**

We evaluated 91 children, of whom 48 were female. In this study sample, the cumulative incidence of epilepsy was 71.4% in the first 24 months, and the main type of seizure was infantile spasms (83.1%). The highest incidence of seizures occurred between 3 and 9 months of age, and the risk remained high until 15 months of age. The incidence of infantile spasms peaked between 4 and 7 months and was followed by an increased incidence of focal epilepsy cases after 12 months of age. Neuroimaging results were available for all children, and 100% were abnormal. Cortical abnormalities were identified in 78.4% of the 74 children evaluated by computed tomography and 100% of the 53 children evaluated by magnetic resonance imaging. Overall, only 46.1% of the 65 children with epilepsy responded to treatment. The most commonly used medication was sodium valproate with or without benzodiazepines, levetiracetam, phenobarbital, and vigabatrin.

**Significance:**

Zika‐related microcephaly was associated with high risk of early epilepsy. Seizures typically began after the third month of life, usually as infantile spasms, with atypical electroencephalographic abnormalities. The seizure control rate was low. The onset of seizures in the second year was less frequent and, when it occurred, presented as focal epilepsy.

**Key Points**

- Epilepsy is a common and early finding in children with Zika‐related microcephaly
- Epileptic encephalopathy with spasms occurred in the majority of cases during the first year of life
- Hypsarrhythmia was not a frequent EEG finding for the epileptic encephalopathy cases; focal epilepsy was the most common type observed in the second year
- Seizure control rate was low

## 1 INTRODUCTION

In 2015, an outbreak of microcephaly was reported in the northeast part of Brazil. Zika virus (ZIKV) was identified as the teratogenic agent, and intrauterine ZIKV infections were observed to have repercussions for the formation and development of the fetal nervous system.^1^, ^2^

To address the public health emergency of congenital Zika syndrome (CZS), the Microcephaly Epidemic Research Group (MERG; http://www.cpqam.fiocruz.br/merg/) was formed in late 2015 and subsequently initiated three epidemiological investigations: a case‐control study of microcephaly, for which the results have been published,^3^, ^4^, ^5^ a cohort study of pregnant women with rash,^6^ and a cohort of children with potential prenatal exposure to ZIKV. In addition, MERG reported on a large case series of children with microcephaly^2^ and proposed an initial description of CZS.^7^

In severe cases, children congenitally infected with ZIKV develop microcephaly, which has a clinical presentation marked by significant craniofacial disproportion as well as severe motor, cognitive, and sensory impairment.^8^, ^9^ Neuroimaging studies have demonstrated that common features of microcephalic children include severe diffuse atrophy of the cerebral volume (especially of the supratentorial compartment), ventriculomegaly, cerebral‐subcortical punctate calcifications, and cortical developmental disorders, primarily lissencephaly.^9^

Of clinical significance, lissencephaly is considered to be a major contributor to pediatric epilepsy cases^10^, ^11^ for which there is an early onset of seizures that are difficult to control with the use of antiseizure medications.^12^ As lissencephaly has been a common finding among the neuroimaging of patients with CZS,^9^, ^12^ it is hypothesized that there may be an increased risk for the occurrence of early epilepsy in children with Zika‐related microcephaly, as suggested in the initial cases series.^13^, ^14^, ^15^, ^16^ Using data collected from a cohort of children born with microcephaly during the 2015‐2016 ZIKV epidemic in northeast Brazil,^2^ this study aims to (1) estimate the incidence of epilepsy during the first 24 months of life, (2) characterize the associated clinical manifestations and electrographic findings, and (3) summarize the treatment responses.

## 2 MATERIALS AND METHODS

The children participating in this study were followed using standardized medical assessments between October 2015 and April 2018 as part of the MERG Children's Bidirectional Cohort based in Recife, Pernambuco, Brazil. The Oswaldo Cruz University Hospital Research Ethics Committee provided ethical approval for this study (Presentation Certificate for Ethical Appreciation—CAAE 52803316.8.0000.5192), and those legally responsible for the infants signed informed consent forms for study participation. All research was conducted in accordance with the Declaration of Helsinki and the codes and regulations of Brazil regarding research on human subjects.

### 2.1 Study participants

The participants in the study included children diagnosed with microcephaly who were born between June 2015 and April 2016 (ie, the period corresponding to the peak of the microcephaly epidemic in Pernambuco state). Participants were classified as confirmed cases of CZS when they had laboratory evidence of ZIKV infection and as highly probable cases when they presented with abnormal neuroimaging findings and negative laboratory results for other congenital infections.^17^

Microcephaly was defined as a head circumference at least two standard deviations below the mean for age and sex; microcephaly cases were further classified as severe with a head circumference at least three standard deviations below the mean.^18^, ^19^ For children born at term (ie, gestational age ≥ 37 weeks), head circumference *z* scores were calculated according to the World Health Organization (WHO) growth chart.^18^ using WHO Anthro software (https://who.int/nutgrowthdb/software/en/). For premature infants, age was corrected in weeks, and head circumference *z* scores were calculated according to the Intergrowth curve.^19^

Infants who underwent only one assessment at a very early age (ie, ≤3 months) were excluded from the analyses.

### 2.2 Participant evaluations

Neurological assessments were performed, according to the study protocol, by the MERG multidisciplinary teams in the neuropediatric outpatient clinic of the Oswaldo Cruz University Hospital (Recife, Pernambuco, Brazil) at the ages of ≤3, 6, 12, 15, 18, 21, and 24 months of life. Additional information for this study was retrieved from outpatient medical records and through interviews with caregivers. Laboratory evaluations for congenital infections were based on the analysis of cerebrospinal fluid (CSF) and/or blood collected during the first days of life and tested by capture‐IgM enzyme‐linked immunosorbent assay for IgM antibodies.^20^, ^21^

All children underwent computed tomography (CT) and/or magnetic resonance imaging (MRI) according to medical indication. The Brazilian Ministry of Health guidelines recommend CT for the initial investigation of children with microcephaly. For those children who did not have MRI by the end of the second year of life, this examination was requested.

The cohort follow‐up included serial electroencephalograms (EEGs), which were performed using the International 10‐20 system, with differentiated montages for neonates at very early ages or participants with a very small head circumference, and with 21 scalp electrodes in those aged >6 months. The digital EEG recordings were obtained during spontaneous sleep, lasting at least 30 minutes, using Neuromap or EMSA Medical Equipment systems, with a sensitivity of 7 or 10 µV/mm and filters of low frequency of 0.5 Hz and high frequency of 70 Hz. The recording speed was 1.5 or 3.0 cm/s according to child age. They were analyzed in standard and/or differentiated bipolar and longitudinal montages for neonates, when appropriate. The type of epilepsy was characterized by the most severe pattern recorded at any time within the period. The occurrence of hypsarrhythmia at any time determined the classification in this modality. A blinded analysis was performed separately by two neurophysiologists certified by the Brazilian Society of Clinical Neurophysiology board. The EEG results were categorized according to the revised glossary of terms most commonly used by clinical electroencephalographers^22^ and classic descriptions of hypsarrhythmia as (1) background rhythm disorganization only; (2) EEG with a focal epileptiform abnormality (EA) pattern (ie, a small area in one hemisphere with epileptiform abnormality)^22^; (3) a multifocal epileptiform abnormality (ie, more than two independent epileptogenic regions present in both hemispheres)^22^; (4) a generalized EA (ie, an abnormality in diffuse bilateral synchronous projection)^22^; (5) hypsarrhythmia (ie, an abnormal pattern with very high‐amplitude slow waves and multifocal spikes and sharp waves in all cortical areas, with disorganized and asynchronous appearance in classic form or increased interhemispheric synchronization, asymmetrical, consistent or predominant single focus, episodes of voltage attenuation or fragmentation, and/or primarily slow activity in variants forms)^22^, ^23^, ^24^; and (6) EA with more than one pattern, usually multifocal and generalized, with a continuous occurrence but without the asynchrony and high‐voltage characteristics defining hypsarrhythmia.^22^ The agreement between the EEG readings by the two neurophysiologists was assessed by kappa coefficient. Discordant analyses were reassessed by joint review, and final classifications were reached by consensus.

### 2.3 Study variables and statistical analyses

The incidence of epilepsy was defined as the age of the child at first seizure. Epilepsy was defined by the occurrence of seizure as reported by the caregiver, recorded in home videos, or witnessed by the attending physician. From these data, the cumulative incidence of epilepsy was evaluated using a Kaplan‐Meier approach. For this method, cumulative incidence was estimated as 1 − *Si*, where *Si* is the cumulative probability of remaining free of seizures, calculated as the product of the conditional survival probabilities for all preceding intervals up to time *i*. For the survival analyses, follow‐up was censored if the participant was lost to follow‐up, died from other causes, or reached the end of the study (ie, 24 months of age). Age‐related patterns in epilepsy prevalence and seizure types were evaluated across the following time periods: 0 to <3 months, 3 to <6 months, 6 to <9 months, 9 to <12 months, 12 to <15 months, 15 to <18 months, 18 to <21 months, and 21‐24 months.

Epilepsy was described by (1) the type of seizure according to the International League Against Epilepsy classifications,^25^ (2) the EEG findings, and (3) the associated use of antiseizure medications (ASMs). To minimize the misdiagnosis of spasms and nonepileptic events, a detailed medical history was undertaken to identify triggers for startle response, behavior before and after events, relationship to sleep and wakefulness, typical events, and the potential for suppression of events by limb restraint or other maneuvers.

Response to antiseizure treatment was defined, for focal epileptic syndrome, as a period of at least 6 months seizure‐free from the seventh month of age^26^ and, for epileptic syndromes with spasms, as a period of at least 28 consecutive days seizure‐free.^27^

## 3 RESULTS

The study included 91 children with microcephaly who were evaluated over the first 2 years of life during a median of four outpatient visits (Table 1). The analytical cohort comprised 48 females and 43 males. Overall, 88 (96.7%) participants were considered to have severe microcephaly. CSF samples from 60 children and blood samples from 23 children were tested for ZIKV; 52 were ZIKV‐IgM+ in CSF and two were ZIKV‐IgM+ in blood. All participants underwent CT and/or MRI neuroimaging (CT & MRI, n = 36; CT alone, n = 38; MRI alone, n = 17;), and 100% of them presented with abnormalities commonly found in congenital ZIKV infection, including corticosubcortical calcifications, cortical developmental disorders with simplification of the gyral pattern (pachygyria/agyria/lissencephaly) or polymicrogyria, and cortical atrophy with ventriculomegaly.

**Table 1:** Clinical, laboratory, and neuroimaging findings among 91 children with Zika‐related microcephaly

Clinical characteristics	Median (P_25_‐P_75_)/patients, n (%)
Evaluations per child	4 (2‐5)
Sex	
Male	43 (47.3%)
Female	48 (52.7%)
Microcephaly	
<−2 SD	3 (3.3%)
<−3 SD	88 (96.7%)
Epilepsy	
Yes	65 (71.4%)
No	26 (28.6%)
Cerebral spinal fluid	
IgM or RT‐PCR positive	53/61 (86.9%)
Brain computer tomography	
Calcifications	68/74 (91.9%)
Ventriculomegaly	66/74 (89.2%)
Simplified gyral pattern	58/74 (78.4%)
Brain magnetic resonance imaging	
Malformation of cortical development	53/53 (100%)
Decreased brain volume	44/48 (91.7%)

**Figure 1:**
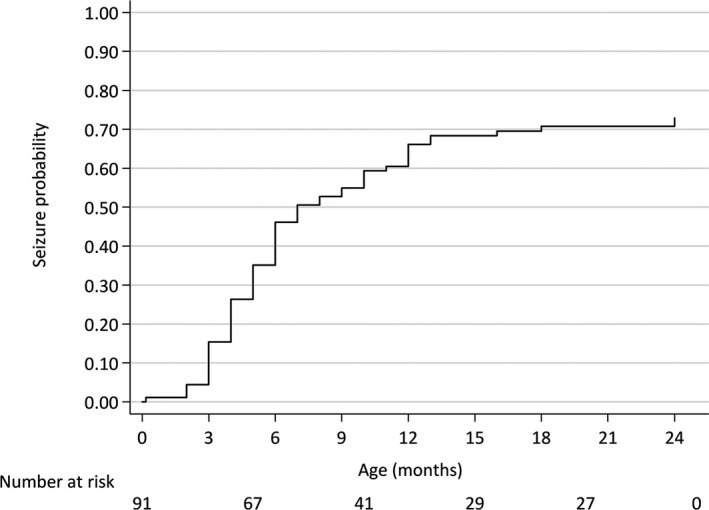
The cumulative incidence of epileptic seizures in the first months of life of infants with microcephaly due to Zika virus

Of the 91 infants included in this study, 65 (71.4%) developed epilepsy during follow‐up (Figure 1) with an incidence of 6.3 cases per 100 person‐months. The earliest case of epilepsy was observed at 5 days of age, and the incidence rate peaked at between 4 and 7 months of age. The median age for first unprovoked seizure was 6 months (interquartile range = 4‐8 months). The incidence of epilepsy by age group and the cumulative incidence are shown in Table 2. By the ninth month of age, 52.7% of participating children had presented with epilepsy, and by the end of the second year, this percentage increased to 71.4%.

**Table 2:** Incidence of epilepsy by age range during the first 24 months of life

Age at onset, mo	Epilepsy/patients followed up	Incidence of epilepsy, %	Cumulative incidence
First year			
0 to <3	4/91	4.4%	—
3 to <6	28/87	32.2%	35.2%
6 to <9	16/59	27.1%	52.7%
9 to <12	7/43	16.3%	60.4%
Second year			
12 to <15	7/36	19.4%	68.1%
15 to <18	1/29	3.4%	69.2%
18 to <21	1/28	3.6%	70.3%
21 to 24	1/27	3.7%	71.4%

The main types of seizure and the most common epileptic syndromes are shown in Table 3. Whereas epileptic spasms were the most common type of seizure in the first year of life, focal seizures predominated in the second year.

**Table 3:** Clinical and EEG characteristics of epilepsy in infants with microcephaly during the first 24 months of life and proportion of infants with seizure control

	Age at onset of epilepsy		
	0 to <3 mo	3 to <12 mo	12‐24 mo
Incidence, n = 91, (%)	4 (4.3%)	51 (56%)	10 (11%)
Type of first seizure	2 focal 1 spasms 1 generalized	3 focal 47 spasms as first seizure^a^ 1 generalized	6 focal^b^ 4 spasms
Epileptic syndrome	1 focal epileptic 1 epileptic encephalopathy with infantile spasms 1 generalized seizure only 1 focal seizure only	2 focal epileptic 49 epileptic encephalopathy with infantile spasms^a^	7 focal epileptic 5 epileptic encephalopathy with infantile spasms^c^
EEG findings	2 focal epileptiform abnormality 2 generalized epileptiform abnormality	2 background rhythm disorganization 26 focal epileptiform abnormality 11 multifocal epileptiform abnormality 10 multifocal generalized plus epileptiform abnormality 2 hypsarrhythmia	7 focal epileptiform abnormality 3 multifocal epileptiform abnormality
Response to treatment at 12 mo	None	10/51 (19.6%) epileptic encephalopathy with infantile spasms	—
Response to treatment at 24 mo	2/4 (50%) focal epileptic	14/51 (27.4%) epileptic encephalopathy with infantile spasms	4/10 (40%): 3 focal epileptic; 1 epileptic encephalopathy with infantile spasms

Abbreviation: EEG, electroencephalographic.

a Two patients had spasms as a second type for a total of 49 spasms cases.

b One patient developed spasms near 12 months, but controlled rapidly. After 3 months, he developed focal epilepsy, which remained uncontrolled at the end of follow‐up.

c One patient had an isolated focal seizure at 2 months and developed spasms at 22 months.

Of the 55 children who had infantile spasms, two had their first seizures classified as isolated focal seizures at 2 and 3 months of age, but then developed new onset infantile spasms at 22 and 10 months, respectively. Two other children had an isolated generalized seizure at 5 days of age and at 3 months of age and then developed infantile spasms at 12 and 6 months, respectively.

Ten children experienced focal epilepsy. One initially had spasm attacks at 12 months, which were promptly controlled with ASM, before developing focal seizures later at 15 months. This child and two others experienced only two seizures prior to control. Two of three children who had weekly seizures responded to treatment. Three children who had daily seizures did not respond to treatment. For one child, seizure frequency was uncertain, but this child was among those who did not respond to treatment.

**Figure 2:**
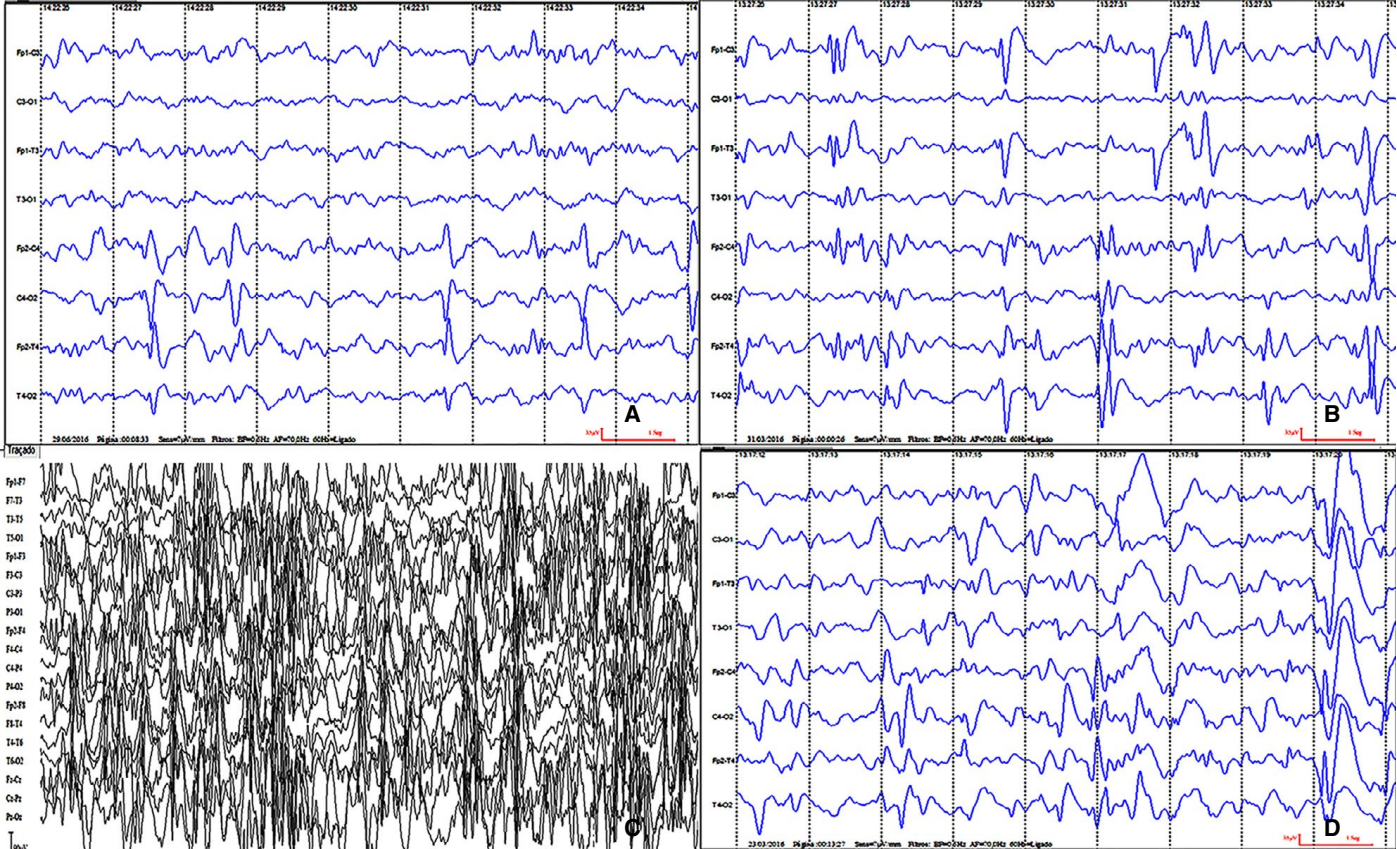
Electroencephalogram (EEG) samples of different patterns of epileptiform abnormality. A, B, C, EEGs acquired using a Neuromap system with the following parameters: high‐frequency filter = 70 Hz; low‐frequency filter = 0.5 Hz; voltage = 7 µV/mm, velocity = 30 mm/segment. D, EEG acquired using an EMSA Medical Equipment system with the following parameters: high‐frequency filter = 70 Hz; low‐frequency filter = 0.5 Hz; voltage = 10 µV/mm, velocity = 30 mm/segment. A, Focal epileptiform abnormality. B, Multifocal epileptiform abnormality. C, Hypsarrhythmia. D, Multifocal generalized plus epileptiform abnormality

In the EEG evaluation, the kappa index for the concordance between the evaluators was 82.9%. Of the 26 nonepileptic participants, the EEGs of 15 were analyzed, of which 13 presented focal or multifocal epileptiform abnormalities and two had no paroxysms (Table 3). Only two of the 65 infants with epilepsy presented with no epileptiform abnormalities, although disorganization of the background rhythms was apparent. Some participants presented several patterns of epileptiform abnormality at different times during the first 2 years of age. Among the 65 epileptic participants evaluated by EEG, the most frequent patterns observed were focal epileptiform abnormalities (53.2%) and multifocal abnormalities (22.8%; Figure 2). An additional 15% experienced multifocal epileptiform abnormalities associated with generalized discharges but without other characteristics of hypsarrhythmia, such as high amplitude, asynchrony, or continuous incidence. Only two participants presented a typical pattern of hypsarrhythmia (Table 3).

The most commonly used antiepileptic drugs were sodium valproate, levetiracetam, benzodiazepines, phenobarbital, and vigabatrin; distribution of use depended in part on availability and/or cost. Treatment was initiated with the minimum recommended daily dosage and was increased weekly or biweekly up to the maximum dose recommended or tolerated (ie, due to the emergence of adverse effects). In children who were <3 months of age, the first treatment regimen was phenobarbital, which was later replaced by sodium valproate in most cases. In children aged ≥3 months, treatment began with valproate alone or in combination with benzodiazepine, followed by levetiracetam and vigabatrin. Carbamazepine and/or lamotrigine were regimens most commonly used in focal seizures. Although vigabatrin is considered the preferential treatment for infantile spasms, only seven patients had access to this medication, due to economic constraints. Adrenocorticotrophic hormone (ACTH) was not used because it has not been made available by the Brazilian Ministry of Health. As CZS is a novel syndrome with uncertainties regarding the viral persistence in the body and brain, steroid therapy in high doses was avoided in these microcephalic children.

Responses to antiseizure treatment were evaluated in two periods (ie, during the first 12 months and by 24 months). In the first period, 51 of the 65 participants with epilepsy were assessed. One child was not evaluated because their seizures began close to the 12th month. In the first period, 10 of 51 (19.6%) evaluated patients achieved seizure control. In the second period, 64 of the 65 participants with epilepsy were assessed, and only 20 (30.8%) responded to antiseizure treatment. Considering both periods, the total rate of response to treatment was 46.1%. The response to treatment was better in patients with focal epilepsy, especially in those cases with early age at seizure onset (Table 3). Epileptic encephalopathy with spasm (ie, West syndrome and spasm without hypsarrhythmia) was refractory to several drug combinations.

## 4 DISCUSSION

In this study, we describe the incidence and characteristics of epilepsy in 91 infants with CZS‐associated microcephaly during the first 2 years of life. Over the duration of follow‐up, 71.4% of the microcephalic children experienced epilepsy, with a peak in incidence between the fourth and seventh months. Spasms were the predominant manifestation of seizure; however, EEG findings were not always typical of West syndrome. After 12 months of age, the incidence of epilepsy decreased, and most new onset seizures were focal in presentation. All children were observed to have abnormal neuroimaging, with notable alterations in the development of the cerebral cortex and varying degrees of lissencephaly (ie, pachygyria/agyria). Only 46.1% of the participants with epilepsy responded to antiseizure treatment by the end of follow‐up.

The hypothesis that Zika‐related microcephaly is associated with a high risk of developing early epilepsy (ie, in the first 24 months of life) was confirmed in this cohort. At the end of 24 months, nearly three‐quarters of the monitored children had epileptic seizures, an incidence similar to that described in other studies of children with extensive lissencephaly.^11^, ^28^ Other recent studies of microcephaly cases associated with congenital ZIKV infections have reported either similar^15^ or lower rates.^12^, ^13^, ^15^, ^16^ Two factors may underlie the difference in rates. The first is the age range assessed. The study by Oliveira‐Filho and colleagues^13^ reported an incidence of 47% in infants monitored in only the first 4 months of life.^12^ As the age range for observation increased, the percentage of affected children increased to 50% in the study by Moura da Silva et al,^12^ which assessed infants up to 8 months, and to 67% in infants assessed up to 1 year of age in the study by van der Linden and colleagues,^15^ which was the similar to our finding at 12 months. Our study confirmed that the highest incidence of epilepsy in children with Zika‐related microcephaly occurs during the first year and provided new insights regarding the development of seizures in the second year.

With regard to the age groups, we hypothesized that a significant proportion of participants would present with early seizures in the first 2 months of life and develop an epileptic encephalopathy in the neonatal period, because lissencephaly, a common finding in these affected infants, is known to be an important cause of Ohtahara syndrome.^10^, ^11^, ^28^ Nevertheless, we did not observe any record of this syndrome in this ZIKV‐affected cohort, a finding consistent with previous studies.^12^, ^13^, ^15^ The largest proportion of epileptic seizures occurred between the third and ninth months of life, with incidence remaining high until the 15th month and very few new cases after this age. This age range is typical for when West syndrome usually develops, regardless of etiology.^29^ We observed epileptic spasms as the main type of seizure, and, even when a given patient presented with another type of associated seizure, spasms were the most significant manifestation. However, the EEG findings recorded in these cases were not typical of West syndrome. In epilepsy associated with lissencephaly, spasms are described as the most common type of seizure, and the presence of focal or multifocal epileptiform abnormality as the most common electrographic pattern,^10^, ^11^, ^30^ which was reported in our population. The spasms described did not always occur in clusters, and there were many isolated events occurring at different times throughout the day, which presented clinically as symmetrical, asymmetrical, or associated with focal signs at the beginning or end. The second most common type of seizure was focal seizures, and these were the predominant type in participants for whom onset of epilepsy occurred after the first year of life. This finding has not previously been reported in the literature. We hypothesize that the observed epilepsy may be related to the severe cortical malformations and calcifications caused by congenital ZIKV infections, because the incidence of early epilepsy is higher in populations with these findings than the general population. We can compare this cohort to children with classical lissencephaly and other congenital infections, such as cytomegalovirus.

Classical lissencephaly is recognized as a neuronal migration disorder. The migration and differentiation of neurons are critical to appropriate electrophysiological functions of the cortex.^28^, ^31^ The incidence of seizures in these cases is approximately 90%, with typical onset before 6 months of age. Experimental ZIKV infection models have demonstrated that ZIKV is a neurotropic virus, with a predilection for neuronal progenitor cells. After infection, ZIKV interferes with cellular differentiation and survival through regulation of genes associated with proliferation and apoptosis.^32^, ^33^, ^34^, ^35^ Thus, congenital ZIKV infection can interrupt the development of neurological tissues, especially the cerebral cortex, reduce the neuronal population, and lead to reduced cortical mantle thickness, areas of brain tissue gliosis and necrosis, and early calcification with appropriate appearance of cytoarchitecture of cortical layers.^32^, ^33^, ^34^, ^35^, ^36^ Consistent with this, neuroimaging studies of CZS cases indicate extensive cortical thickness and abnormal patterning of sulci and gyri on the surface of the brain. All of these abnormalities may be involved in the development of early Zika‐related epilepsy. In lissencephalies of the other causes, especially those of genetic origin, there is a similar abnormal neuronal migration, with loss of cortical architecture and macroscopic simplification of the cortical mantle; however, gliosis and calcifications are not observed.^28^, ^31^ We hypothesize that these differences may alter clinical manifestations, at least in early ages, during which the onset of Zika‐related seizures was not as frequent. Because the participants were primarily severe microcephalic cases with major neuroimaging abnormalities, the correlation between imaging findings and the risk of specific epileptic syndromes could not be evaluated in this study.

Other congenital infections are also associated with early epilepsy. Individuals with congenital cytomegalovirus infections appear to present a clinical picture similar to CZS cases, with overlapping clinical and neuroimaging features. The incidence of congenital cytomegalovirus‐associated epilepsy has been reported to vary between 10% and 56%,^37^ and the emergence of seizures has been linked to disorders of cortical development, ventriculomegaly, and calcifications. In contrast with CZS cases, focal seizures are the most common type associated with congenital cytomegalovirus infections.^37^, ^38^ As the developmental cortical disorders in the current ZIKV cohort were severe and extensive, this finding may, in part, explain the observed higher incidence and greater severity of epilepsy relative to published reports on congenital cytomegalovirus‐associated epilepsy.^37^

The role of ZIKV in epileptogenesis is not yet fully understood, but it is well established that congenital infections can lead to structural abnormalities in brain tissue. We consider that the involvement of the virus in cellular division mechanisms of infected neurons may predispose to changes in cellular electrophysiology, making them hyperexcitable.

In relation to the EEG findings, it should be noted that a very small number of participants with spasms presented a trace of hypsarrhythmia at some point during the follow‐up. In the vast majority, however, we observed the presence of focal epileptiform abnormality in one or more areas accompanied by disorganization of the background cerebral rhythms. In some cases, the epileptiform abnormality was more varied and multifocal, and generalized paroxysms were observed in the same EEG. Extensive cortical abnormalities with significant simplification of the grooves and ridges and a reduction in the parenchymal volume and neuronal population likely provide the anatomical substrate for these findings.^39^ Cortical reduction and simplification interfere in the neurophysiology of brain tissue, affecting both background brain activity and the generation and amplitude of nonphysiological potentials, resulting in a pattern of focal epileptiform abnormality with less amplitude, and reduced propagation and synchronization of epileptiform paroxysms.^39^

The overall response rate to treatment was poor, with approximately 20% seizure control in the first year and 30% in the second year. The low response rate may have been influenced by the medications used, as not all participants had access to recommended treatments for epileptic spasms, such as vigabatrin, ACTH, and corticosteroids. However, epileptic spasms associated with lissencephaly are often refractory to medical treatment, including the recommended medications. In a separate study of children with Zika‐related microcephaly, the response rate to vigabatrin was also reported to be low (22%).^10^

The treatment response rate may have been influenced by the following factors. First, selection bias may have been introduced, as the presence of epileptic seizures may have increased adherence to medical follow‐up. Nevertheless, any potential impact of this bias is mitigated in that all of the participants were microcephalic and thereby symptomatic with neurological impairments, a presentation that favors good adherence to the follow‐up regardless of epilepsy status. Overall, the incidence rate reported here appears to be consistent with previously published rates and is a plausible estimate for populations of children with Zika‐related microcephaly. A second limitation is that the method of diagnosing seizures in our study may have been subject to misclassification bias. The affected infants may have had subtle seizures, which may not have been recognized by the family, or abnormal motor events that may have been erroneously reported as seizures. Nevertheless, the detailed history of paroxysmal event used here is the primary basis for the diagnosis of epilepsy. In addition, the use of home videos was useful for minimizing misclassification. In medical practice, not all epileptic patients will undergo video EEG evaluation, as had occurred in the infants included in this study.

## 5 CONCLUSION

In conclusion, Zika‐related microcephaly is associated with a high risk of developing epilepsy in the first 2 years of life, with an incidence rate of 6.3 per 100 person‐months. The highest proportion of incident cases (ie, new onset seizures) was observed between the fourth and seventh months of life, the most common type of seizure was spasm epileptic without hypsarrhythmia, and the rate of seizure control was low. The probability of developing epilepsy decreased after 12 months, and thereafter, most commonly presented as structural focal epilepsy, with a low rate of response to treatment.

## CONFLICT OF INTEREST

None of the authors has any conflicts of interest to disclose. We confirm that we have read the Journal's position on issues involved in ethical publication and affirm that this report is consistent with those guidelines.
